# The Gradual Cooling of the Earth

**Published:** 1883-08

**Authors:** 


### THE GRADUAL COOLING OF THE EARTH.

In a "Treatise on Natural Philosophy," by Professors Sir W. Thomson and P. G. Tait, Sir W. Thomson, speaking of an opinion advanced by Sir Charles Lyell, respecting the possible maintenance of the earth's heat without change throughout countless ages, used words which, says *Knowledge,* may be applied without change of a word to the stupendous theory advanced by Sir C. Siemens not so very long since—such an idea of a practically endless cycle "violates the principles of natural philosophy in exactly the same manner, and to the same degree, as to believe that a clock constructed with a self-winding movement may fulfil the expectations of its ingenious inventor by going forever." The earth is necessarily cooling from century to century; her volcanic energies are certainly diminishing, as certainly, to use an illustration of Sir W. Thomson's, as the quantity of gunpowder in a "monitor" is diminishing when hour after hour she is seen to discharge shot and shell, whether at a nearly equable rate or not, without receiving fresh supplies of ammunition.

